# Electrospun PLA/PCL Membranes for Sustained Transdermal Rifampicin Delivery: Biocompatibility, Stability, and Antimycobacterial Activity

**DOI:** 10.3390/polym18151814

**Published:** 2026-07-24

**Authors:** Esmeralda Juárez, Elizabeth Ortiz, Ningel Omar Gama, Andy Ruiz, Silvia Guzmán-Beltrán, Wendy Arias, Miguel Angel Aguilar-Méndez, Eduardo San Martin-Martínez, Horacio Vieyra

**Affiliations:** 1 Laboratorio de Alta Contención Biológica, Instituto Nacional de Enfermedades Respiratorias Ismael Cosío Villegas, Calz. de Tlalpan 4502, Sección XVI, Tlalpan, Ciudad de México 14080, Mexico; ejuarez@iner.gob.mx (E.J.); andy.ruiz@iner.gob.mx (A.R.); 2 Tecnológico de Monterrey, Escuela de Ingeniería y Ciencias, Eduardo Monroy Cárdenas 2000, San Antonio Buenavista, Toluca de Lerdo 50110, Mexico; a01368747@tec.mx; 3 Centro de Investigación en Ciencia Aplicada y Tecnología Avanzada, Instituto Politécnico Nacional, Legaria 694, Colonia Irrigación, Ciudad de México 11500, Mexico; ngamac1700@alumno.ipn.mx (N.O.G.); wen.ariasb@gmail.com (W.A.); maguilarme@ipn.mx (M.A.A.-M.); 4 Departamento de Investigación en Microbiología, Instituto Nacional de Enfermedades Respiratorias Ismael Cosío Villegas, Calz. de Tlalpan 4502, Sección XVI, Tlalpan, Ciudad de México 14080, Mexico; sguzman@iner.gob.mx

**Keywords:** poly(ε-caprolactone), polylactic acid, electrospinning, biomedical patches, tuberculosis, rifampicin, drug delivery systems

## Abstract

Poor adherence to prolonged antibiotic regimens remains a major challenge in the treatment and prevention of chronic infectious diseases such as tuberculosis. Transdermal drug delivery systems capable of sustained antibiotic release may improve therapeutic compliance while reducing the need for frequent oral administration. In this study, electrospun polymeric membranes based on poly(lactic acid) (PLA) and poly(ε-caprolactone) (PCL) were developed as transdermal rifampicin delivery platforms. Homogeneous nanofibrous membranes with average fiber diameters of approximately 250 nm were successfully fabricated and exhibited efficient drug incorporation while preserving the structural integrity of the polymeric matrix. The electrospun membranes retained sufficient tensile strength and dimensional stability after accelerated temperature–humidity aging, supporting their stability during storage, handling, and application. In vitro cytotoxicity and biocompatibility assays using primary human peripheral blood mononuclear cells (PBMCs) demonstrated that the developed systems did not induce significant cytotoxic or pro-inflammatory responses. Transdermal permeation studies using an in vitro mouse skin model demonstrated sustained rifampicin diffusion for at least 72 h. Importantly, the antibiotic recovered after skin permeation preserved antimycobacterial activity against *Mycobacterium tuberculosis* H37Ra and *Mycobacterium bovis* BCG, confirming that rifampicin maintained its biological functionality after electrospinning and transdermal migration. Overall, these findings demonstrate the potential of electrospun PLA/PCL membranes as stable and biocompatible transdermal antibiotic delivery systems capable of sustained release and preservation of antimicrobial activity. This proof-of-concept study supports the translational potential of electrospun polymeric platforms for controlled antibiotic delivery in long-term infectious disease therapies.

## 1. Introduction

Antimicrobial resistance (AMR) is a major global public health challenge. International organizations such as the World Health Organization (WHO), the Pan American Health Organization (PAHO), and the Centers for Disease Control and Prevention (CDC) have reported a sustained increase in multidrug-resistant (MDR) bacteria, including strains resistant to last-generation antimicrobial agents. The most prevalent bacterial infectious diseases worldwide are respiratory, urinary, and intestinal tract infections [1,2]. AMR also represents a substantial economic burden. According to the World Bank report entitled Drug-Resistant Infections: A Threat to Our Economic Future, antimicrobial resistance could lead to economic losses of up to 5% of the gross domestic product in developing countries [3,4]. AMR is also associated with an increasing number of deaths [5].

Although antibiotic resistance is a natural evolutionary phenomenon, it has been markedly accelerated by the inappropriate and indiscriminate use of antibiotics over time [6,7,8]. A recent example is the excessive and often unnecessary use of antibiotics during the early stages of the SARS-CoV-2 (COVID-19) pandemic, which has contributed to the emergence and spread of antibiotic resistance and will likely have long-term consequences for global health [9]. Therefore, there is an urgent need not only for new antimicrobial agents, but also for improved public health policies promoting the rational use of antibiotics and for innovative therapeutic strategies that enable more efficient use of existing drugs [10,11].

Poor adherence to treatment is a major driver of multidrug antibiotic resistance. The main reasons for treatment discontinuation include forgetfulness, perceived clinical improvement, and concern about drug-related adverse effects [12,13]. In this context, nanomedicine offers alternative drug delivery strategies that maintain controlled, therapeutically effective drug concentrations without the need for daily oral administration. One promising approach involves the use of polymeric nanofiber-based dressings in the form of transdermal patches, in which antimicrobial agents are incorporated within the fibrous matrix and subsequently delivered through the skin [14]. Such systems may reduce the need for conventional dosing regimens and avoid the oral route, thereby minimizing drug degradation caused by variations in gastric and intestinal pH and reducing systemic adverse effects [15,16].

One method for fabricating therapeutic patches is electrospinning, a nanofiber production technique in which a polymeric solution is processed to form nanoscale fibers, making it particularly suitable for drug-delivery applications [17,18,19]. Biodegradable polymers such as polylactic acid (PLA), poly(ε-caprolactone) (PCL), and polyvinyl alcohol (PVA) have been widely investigated as drug delivery matrices for the incorporation of therapeutic agents, including antibiotics such as gentamicin, vancomycin, amoxicillin, and ciprofloxacin [20,21,22,23]. In this study, polymeric nanofiber-based dressings were developed by electrospinning using a PLA/PCL matrix for the sustained transdermal delivery of rifampicin, a first-line antibiotic used in the treatment of tuberculosis.

Tuberculosis (TB) is the leading cause of death attributable to a single infectious agent in the world. In 2023, approximately 10.8 million new cases and 1.25 million deaths were reported, in addition to 176,000 cases of multidrug-resistant or rifampicin-resistant tuberculosis (MDR/RR-TB), highlighting the direct impact of poor treatment adherence and incomplete therapeutic regimens [24]. Despite global efforts and the increasing treatment success rate in drug-susceptible TB, substantial gaps remain with respect to international goals established for the coming years. Among the main factors limiting treatment adherence are the prolonged duration of therapeutic regimens and the adverse effects associated with antibiotic-related toxicity [24,25].

Rifampicin, a lipophilic drug, was selected as a model compound due to its clinical relevance in the treatment of tuberculosis. As a first-line antibiotic against *Mycobacterium tuberculosis*, rifampicin is widely used in standard therapeutic regimens. Rifampicin is associated with hepatotoxicity and nephrotoxicity, which frequently lead to discontinuation of therapy and can lead to drug resistance, prompting research for strategies to reduce rifampicin toxicity [26,27,28]. Transdermal delivery of rifampicin has been proposed as a strategy to improve the drug’s bioavailability while reducing side effects [29,30,31], making it a suitable candidate for evaluating the incorporation of rifampicin into electrospun polymeric dressings to achieve controlled transdermal release.

Rifampicin, a lipophilic drug, was selected as a model compound due to its clinical relevance in the treatment of tuberculosis. As a first-line antibiotic against *Mycobacterium tuberculosis*, rifampicin is widely used in standard therapeutic regimens, making it a suitable candidate for evaluating its incorporation into electrospun polymeric dressings and its controlled transdermal release. In this study, rifampicin-loaded dressings were fabricated and characterized for morphology, mechanical properties, and drug release behavior. Cytotoxicity and biocompatibility were assessed using primary human leukocytes, while drug release was quantified by chromatographic techniques. The biological performance of the system was further evaluated in vitro using a proof-of-concept assay in a mouse skin model to assess transdermal drug migration and the antimicrobial activity of released rifampicin. Overall, this approach enabled the evaluation of these dressings as controlled drug-delivery devices with the potential to reduce poor treatment adherence by providing sustained antibiotic delivery.

## 2. Materials and Methods

### 2.1. Materials

Poly(ε-caprolactone) (PCL, Mw ≈ 80,000) and polylactic acid (PLA, Mw: 116,000 g/mol) were selected as polymeric matrices due to their biocompatibility, biodegradability, and suitability for electrospinning processes. PCL and PLA (Ingeo™ Biopolymer 3052D) were purchased from Sigma-Aldrich (St. Louis, MO, USA) and NatureWorks (Plymouth, MN, USA), respectively. Sulfuric acid (H_2_SO_4_, analytical grade, 98 wt%) and nitric acid (HNO_3_, analytical grade, 60 wt%) were obtained from Sigma-Aldrich (St. Louis, MO, USA) and used as auxiliary reagents. Acetone and ethanol (analytical grade) were purchased from Meyer (Mexico City, Mexico), while N,N-dimethylformamide (DMF) was obtained also from Sigma-Aldrich.

### 2.2. Drug Preparation

Rifampicin (RIF) was purchased from Sigma-Aldrich (R3501). Solubility tests were performed using 1 mL of a DMF/acetone mixture (50:50, *v*/*v*). Rifampicin was added incrementally at 10 mg every 5 min, with constant stirring at 650 rpm, until the solution was saturated. The assays were initially conducted at room temperature, and, when necessary, the temperature was gradually increased to 50 °C to facilitate dissolution [22,32]. Rifampicin showed a solubility of up to 200 mg/mL under these conditions. Therefore, a concentration of 25 mg of RIF per 3 mL of polymeric solution was selected to ensure solution stability and proper nanofiber formation during electrospinning.

### 2.3. Preparation of Polymeric Solutions

A solvent mixture of N,N-dimethylformamide (DMF) and acetone (50:50, *v*/*v*) was prepared, in which polylactic acid (PLA) and poly(ε-caprolactone) (PCL) were dissolved at a concentration of 10% (*w*/*v*). Rifampicin (RIF) was subsequently incorporated into the polymeric solution. The dissolution process was carried out under magnetic stirring (300–700 rpm) at 30–60 °C for 2–4 h, followed by continued mixing at 50–80 °C until complete homogenization was achieved. Prior to electrospinning, all polymeric solutions were loaded into 5 mL syringes and degassed using an ultrasonic bath (Elma D-78224, Singen, Germany) for 15 min (25 kHz, continuous mode, 40 W, 25–30 °C). The selected concentrations of PLA (10% *w*/*v*), PCL (10% *w*/*v*), and rifampicin were established after preliminary electrospinning trials aimed at identifying formulations with adequate viscosity, solution stability, and continuous fiber formation. These concentrations provided stable electrospinning conditions and homogeneous nanofibrous membranes with minimal bead formation and were therefore selected for the subsequent physicochemical and biological evaluations. The mix was prepared to generate a dressing containing 10 mg of rifampicin.

### 2.4. Electrospinning Process for Dressing Fabrication

The polymeric solution was loaded into the syringe pump of a FLUIDNATEK LE 100 electrospinning system (Bioinicia, Valencia, Spain) and dispensed at a flow rate of 1200 µL/h. A voltage of 12 kV was applied to generate the electrospinning jet, while a tip-to-collector distance of 15 cm was established to promote fiber elongation and nanofiber deposition onto the metallic collector. Nanofibers were collected on a 30 cm^2^ square stainless-steel collector. Electrospinning was carried out for 6 h, producing nonwoven membranes with an average thickness of approximately 0.26 mm. Upon completion of the process, the electrospun dressings were removed from the collector and dried in an oven at 38 °C for 1 h. The samples were subsequently sterilized by ultraviolet-C (UVC) irradiation for 10 min.

#### Determination of Rifampicin Loading Efficiency

To determine RIF loading efficiency on the nanofibers, the HPLC-DAD (Agilent Technologies, Santa Clara, CA, USA) method was used. For this purpose, samples of the RIF standard (Cat. No. R3501, Sigma-Aldrich) and circular samples of the PLA/PCL-RIF nanofibers, 1 cm in diameter, were used; these were solubilized in HPLC-grade acetonitrile (Cat. No. A998-4, Fisher Scientific, Waltham, MA, USA). The samples were placed in an ultrasonic bath (10 min, 25 kHz, 25 °C, 100% power) and filtered through a membrane with a pore diameter of 0.22 µm.

A mobile phase (consisting of an acetonitrile/buffer solution (0.05 M potassium monobasic phosphate) adjusted to pH 3.7 with phosphoric acid (38:62)) was used, filtered through a PVDF membrane with a pore diameter of 0.22 µm. The sample injection volume was 5 µL, with a flow rate of 1 mL/min, the column at 25 °C, and a detection wavelength of 335 nm. A calibration curve was used with an injection of 5 µL, in a concentration range of 25–400 µg/mL.

### 2.5. Morphological Characterization of Dressings by Scanning Electron Microscopy (SEM)

The surface morphology and fiber diameter of the electrospun dressings were evaluated by scanning electron microscopy (SEM). Analyses were performed using a JSM-6390LV microscope (JEOL, Peabody, MA, USA). Prior to imaging, samples were coated with a thin conductive gold layer using a sputtering system (Desk IV, Denton Vacuum) to minimize charging effects during analysis. Micrographs were acquired at different magnifications using an accelerating voltage of 20 kV. Fiber diameters were determined from the obtained micrographs through image analysis using ImageJ 1.x software (Rasband, W.S., ImageJ, U.S. National Institutes of Health, Bethesda, MD, USA).

### 2.6. Functional Group Analysis by Fourier Transform Infrared Spectroscopy (FTIR)

The identification of functional groups and the evaluation of potential interactions among the components of the electrospun dressings were performed using Fourier transform infrared spectroscopy (FTIR). Analyses were performed using a Cary 630 spectrometer (Agilent Technologies, Santa Clara, CA, USA) equipped with an attenuated total reflectance (ATR) accessory fitted with a single-reflection diamond crystal. Spectra were recorded over a wavenumber range of 4000–600 cm^−1^, with a resolution of 4 cm^−1^ and 32 scans averaged per sample. The assignment of the characteristic absorption bands was performed by comparison with previously reported FTIR spectra of PLA, poly(ε-caprolactone), and rifampicin available in the scientific literature.

### 2.7. X-Ray Diffraction Analysis of Nanofibers

X-ray diffraction (XRD) measurements were performed to evaluate the structural ordering of the polymeric nanofibers. Samples were cut into 10 mm × 10 mm sections prior to analysis. Diffraction patterns were obtained using CuKα radiation (λ = 1.5418 Å), operated at 30 kV and 20 mA. Data were collected over a 2θ range of 5–60°, using a step size of 0.05° and a counting time of 15 s per step. Each sample was mounted on a 900 mm^2^ aluminum sample holder (30 mm × 30 mm) for analysis. The diffraction peaks were identified and interpreted by comparison with previously reported X-ray diffraction patterns of poly(lactic acid), poly(ε-caprolactone), and rifampicin-containing electrospun polymeric systems available in the scientific literature.

### 2.8. Accelerated Weathering Test Under Temperature–Humidity Cycling in a Climatic Chamber

Accelerated weathering tests were performed to evaluate the long-term mechanical stability of the electrospun PLA/PCL membranes under severe environmental conditions that may be encountered during storage, transportation and handling. Although the membranes are intended for transdermal application at physiological temperatures (approximately 32–37 °C), the accelerated aging protocol defined in IEC 60068-2-38:2009 [33] was selected to promote thermally and moisture-induced degradation mechanisms within a shortened experimental period. The selected conditions (65 °C and 93% relative humidity) accelerate moisture uptake, hydrolytic degradation of the polyester matrix, molecular chain relaxation, and changes in the fibrous microstructure, providing an assessment of the structural integrity and mechanical stability of the electrospun membranes [34,35].

Accelerated weathering tests were performed using a CSZ ZPH32 environmental chamber (Cincinnati, OH, USA) under standard temperature–humidity cycling conditions according to the International Electrotechnical Commission protocol IEC 60068-2-38:2009. Each humid heat cycle consisted of two subcycles. The first subcycle started at 25 °C and 50% relative humidity (RH), followed by exposure at 65 °C and 93% RH for 4 h, then 25 °C and 93% RH for 0.75 h, followed again by 65 °C and 93% RH for 4 h, and finally 25 °C and 93% RH for 8 h. Temperature ramping between stages required 1.75 h for each heating or cooling transition, and an additional 0.25 h was required to return to ambient temperature. The second subcycle started at 25 °C and 40% RH. Samples were exposed to 65 °C and 93% RH for 4 h, followed by 25 °C and 93% RH for 45 min, then again to 65 °C and 93% RH for 4 h, and finally to 25 °C and 93% RH for 1.75 h. Heating and cooling transitions also required 1.75 h each. Subsequently, the dressings were gradually cooled to −10 °C under uncontrolled RH conditions and maintained at this temperature for 3.5 h, followed by 1.25 h at 25 °C. Cooling to −10 °C required 30 min, while reheating to 25 °C required 1 h. Each complete cycle lasted 2 days and was repeated five times, resulting in a total test duration of 10 days. After environmental exposure, the polymeric dressings were conditioned at 25 °C for an additional 7 days under laboratory conditions prior to characterization.

### 2.9. Tensile Mechanical Testing of the Dressings

The mechanical properties of the electrospun dressings were determined by uniaxial tensile testing according to ASTM D882-10, using a TA-XT2i texture analyzer (Stable Micro Systems, Godalming, UK) equipped with a 25 N load cell. Tensile tests were performed on PLA/PCL and PLA/PCL/RIF dressings, both in their initial condition and after exposure to the temperature–humidity climatic chamber test. Samples were cut into rectangular strips with a nominal width of 10 mm. The length, thickness, and cross-sectional area of each strip were measured individually prior to testing, accounting for dimensional variations induced by environmental conditioning. The gauge length was set to approximately 40 mm. Specimens were mounted between the grips and tested at a constant crosshead speed of 0.5 mm/s until failure. Three specimens were evaluated for each formulation and condition. From the resulting stress–strain curves, Young’s modulus (E), ultimate tensile strength (UTS), stress at break (σb), and elongation at break (εb) were calculated. Results are reported as mean ± standard deviation.

### 2.10. Cytotoxicity and Biocompatibility Evaluation in Human Cells

The cytotoxicity and biocompatibility of the polymeric nanofiber-based dressings were evaluated using primary cultures of peripheral blood mononuclear cells (PBMCs) obtained from leukocyte-rich buffy coats collected from healthy donors at the blood bank of the National Institute of Respiratory Diseases (INER, Mexico City, Mexico). The institutional review board waived informed consent on the grounds of donor anonymity. PBMCs from seven donors were isolated by density gradient centrifugation using Lymphoprep (Axis Shield, Norton, MA, USA) and resuspended in RPMI-1640 medium (Gibco, Waltham, MA, USA) supplemented with 200 nM glutamine (Gibco) and 10% heat-inactivated human serum (Valley Biomedical, Winchester, VA, USA). Cells were seeded at a density of 1 × 10^6^ cells per well in 24-well culture plates. A 1 cm^2^ section of either drug-free PLA/PCL dressings or rifampicin-loaded PLA/PCL dressings (PLA/PCL–RIF) was added to each well. Cultures were incubated at 37 °C in a humidified atmosphere containing 5% CO_2_ for 24 h to assess early cytotoxicity and biocompatibility. Untreated cells were included as a negative control (MEDIUM), while a separate well stimulated with 200 ng/mL lipopolysaccharide from *Escherichia coli* (LPS, Sigma-Aldrich) served as a positive control for inflammatory activation. After 24 h of incubation, cell morphology was documented by optical microscopy, and culture supernatants were collected and cryopreserved at −20 °C for subsequent quantification of pro-inflammatory cytokines. Fresh medium was then added to the wells, and cultures were maintained for an additional 6 days, for a total incubation period of 7 days, to evaluate long-term cytotoxic or pro-inflammatory effects. At the end of the 7-day culture period, supernatants were collected again and stored at −20 °C for further analysis. Cells were recovered, and viability was determined using the trypan blue exclusion assay (Gibco).

### 2.11. Cytokine Quantification

The cytokines IL-2, IL-4, IL-6, IL-8, IL-10, TNF-α, IFN-γ, and GM-CSF were quantified in culture supernatants using an 8-plex human cytokine kit (Bio-Rad, Hercules, CA, USA), following the manufacturer’s instructions.

### 2.12. In Vitro Transdermal Diffusion Assays Using a Skin Model

The transdermal diffusion of rifampicin was evaluated using an in vitro mouse skin model as a proof-of-concept assay. Abdominal skin was obtained from two BALB/c mice maintained under standard animal facility conditions and euthanized in accordance with protocols approved by the Institutional Committee for the Care and Use of Laboratory Animals (CICUAL) of INER (code B10-25). After euthanasia, the abdominal area was shaved, and the skin was excised into 1 cm^2^ sections. Each skin fragment was placed onto polycarbonate membranes (0.4 µm pore size) fitted into 12-well culture plates containing 2 mL of RPMI 1640 medium supplemented with 200 nM glutamine and 10% heat-inactivated fetal bovine serum. The polycarbonate insert served as a physical interface between the tissue and the culture medium. A 1 cm^2^ PLA/PCL dressing containing the previously calculated drug load was placed on top of each skin fragment. Skin samples without dressings and drug-free PLA/PCL dressings were included as controls. The experimental systems were incubated at 37 °C in a humidified atmosphere containing 5% CO_2_. Culture medium was completely collected after 24 h and replaced with fresh medium. This procedure was repeated to obtain culture media at 48 and 72 h. The collected media were stored at −20 °C until further analysis to quantify the drug diffused through the skin and assess its antimicrobial activity.

### 2.13. Preparation of Mycobacterium tuberculosis and M. bovis BCG Stock Cultures

*Mycobacterium tuberculosis* H37Ra and *Mycobacterium bovis* BCG Danish strain were obtained in lyophilized form from the American Type Culture Collection (ATCC; products 25177 and 35733, respectively). The bacteria were reactivated and cultured for 21 days in Middlebrook 7H9 broth supplemented with glycerol and ADC enrichment to generate bacterial stocks for the evaluation of rifampicin’s antibiotic activity in the skin model. Bacterial concentration was determined by the colony-forming unit (CFU) method.

### 2.14. Evaluation of the Antibiotic Activity of Released Rifampicin

The antibiotic activity of released rifampicin was evaluated using the resazurin microtiter assay (REMA), a colorimetric method that quantifies mycobacterial growth [36]. In this assay, culture medium collected from the transdermal system was used as the source of the antibiotic. Briefly, 96-well microplates were prepared by adding 100 µL of a bacterial suspension in the logarithmic growth phase of *Mycobacterium tuberculosis* H37Ra or *Mycobacterium bovis* BCG. Subsequently, 100 µL of culture medium containing the released rifampicin was added, either undiluted or at 1:50 and 1:100 dilutions. Plates were incubated for 5 days at 37 °C in a humidified atmosphere containing 5% CO_2_. After the incubation period, 0.025% resazurin (Sigma-Aldrich) was added to each well, and the plates were incubated for an additional 24 h to assess bacterial growth inhibition. A rifampicin solution at 200 ng/mL (Sigma-Aldrich) was used as a positive control. A color change from blue to pink indicated bacterial growth.

### 2.15. Chromatographic Quantification of Rifampicin in Transdermal Diffusion Samples

The amount of rifampicin (RIF) permeating through the skin during the transdermal diffusion assay was quantified by HPLC-DAD, as described above. Standard RIF solutions and culture medium samples collected from PLA/PCL (control) and PLA/PCL–RIF treatments were extracted with HPLC-grade acetonitrile and analyzed in triplicate. The injection volume was 20 µL, the flow rate was 1 mL/min, the column temperature was 25 °C, and detection was at 335 nm. The amount of RIF detected in 2 mL of culture medium collected at 24, 48, and 72 h after patch application was calculated directly from the calibration curve. The percentage of rifampicin permeated through the skin was determined as the ratio of the quantified amount of RIF in the medium to the theoretical amount of RIF in the patch sample. PLA/PCL dressings without rifampicin were used as negative controls to rule out chromatographic interference, yielding a consistent zero signal.

### 2.16. Statistical Analysis

Repeated-measures one-way ANOVA was performed for cell viability data after confirmation of normality using the Shapiro–Wilk test. Dunnett’s test was applied for post hoc multiple comparisons. Cytokine production data, which did not follow a normal distribution, were analyzed using the Wilcoxon rank-sum test for pairwise comparisons. A value of *p* < 0.05 was considered statistically significant. Statistical analyses were performed with Prism version 11 for Mac (GraphPad Software, San Diego, CA, USA).

## 3. Results and Discussion

### 3.1. Morphological Characterization of the Dressings by SEM

**Figure 1:**
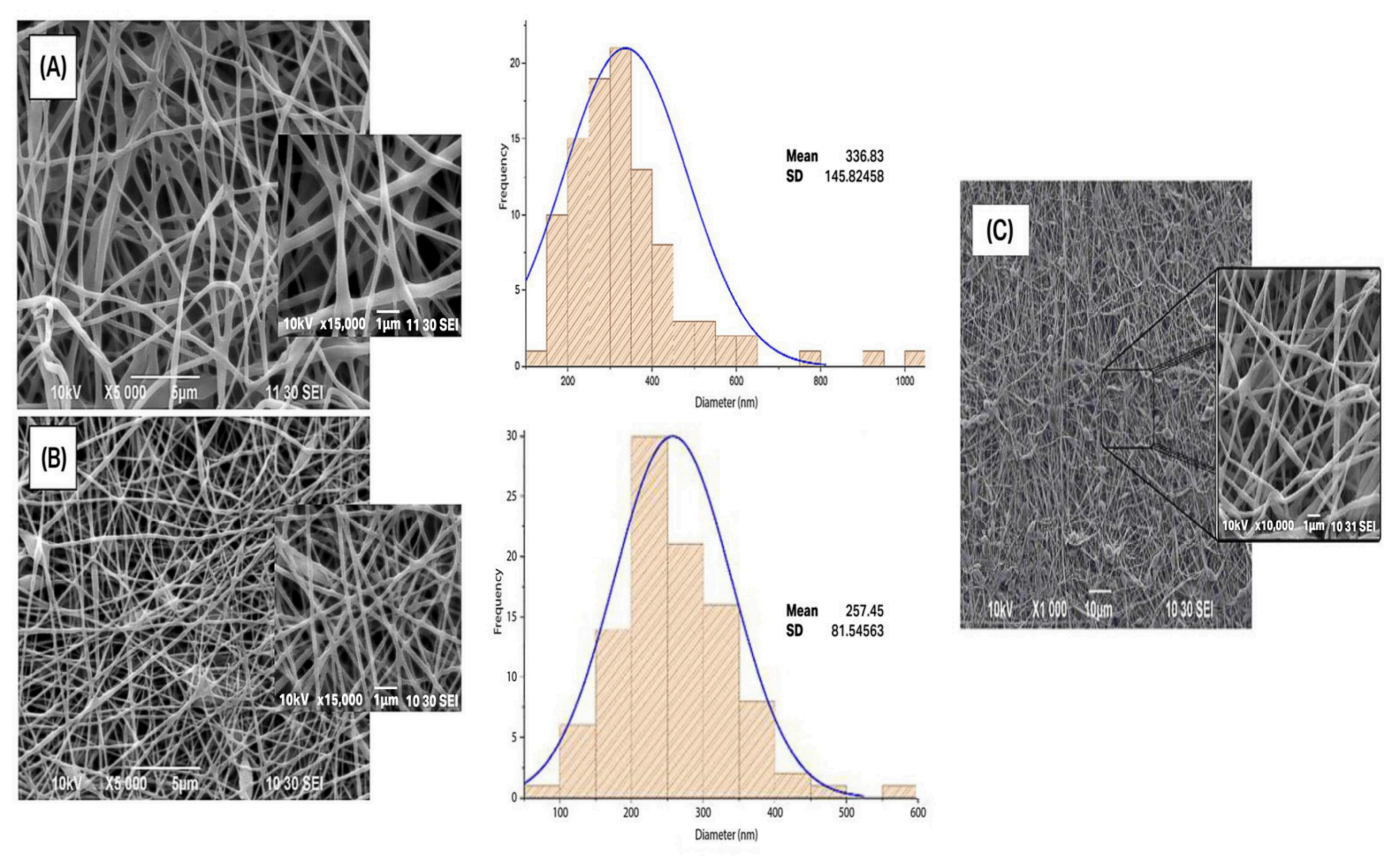
SEM micrographs and fiber diameter distributions of electrospun nanofibers: (**A**) Drug-free PLA/PCL dressing. (**B**) Rifampicin-loaded PLA/PCL dressing (PLA/PCL–RIF). (**C**) Rifampicin-loaded PLA/PCL dressing (PLA/PCL–RIF) at low magnification.

PLA/PCL and PLA/PCL–RIF nanofiber samples, corresponding to drug-free and rifampicin-loaded dressings, respectively, were characterized by SEM (Figure 1A,B). SEM micrographs revealed continuous fibrous structures in both formulations, with clear differences in fiber morphology and average diameter. Rifampicin-loaded nanofibers exhibited a smaller average fiber diameter (257 nm) compared with the drug-free PLA/PCL nanofibers (336 nm). This reduction in fiber diameter may be associated with the presence of ionizable functional groups in rifampicin, such as carbonyl, amino, and hydroxyl groups, which could increase the stretching of the polymer jet under the applied electric field during electrospinning. Morphological analysis further revealed differences in fiber organization between the two formulations. Drug-free PLA/PCL dressings exhibited a more interconnected fibrous network, with a higher degree of interfiber junctions, whereas PLA/PCL–RIF nanofibers appeared smoother and more individualized, with fewer fused regions. Although some bead-like structures were observed in the rifampicin-loaded formulation, the overall fibrous morphology remained homogeneous and suitable for dressing fabrication (Figure 1C) [37,38].

Overall, SEM analysis showed that the evaluated formulations formed continuous nanofibrous networks, with clear differences in defect content, degree of interfiber fusion, and fiber diameter dispersion. These structural features are particularly relevant, as they may directly influence the mechanical performance of the dressings and the release behavior of the incorporated drug [39,40].

Electrospinning offers several advantages over other processing techniques, such as phase separation or self-assembly, including its ability to produce nanofibers with tunable diameters and morphologies using relatively simple equipment, at low cost and with high production rates [41,42]. In this context, polyester-based systems such as PLA and PCL have been widely used to fabricate drug-loaded nanofibers, including formulations containing antibiotics such as gentamicin [43], vancomycin [44], and amoxicillin [23], with promising outcomes. The present results are consistent with these reports and support the suitability of electrospun PLA/PCL dressings as potential transdermal drug delivery platforms.

### 3.2. Fourier Transform Infrared Spectroscopy (FTIR) Analysis

**Figure 2:**
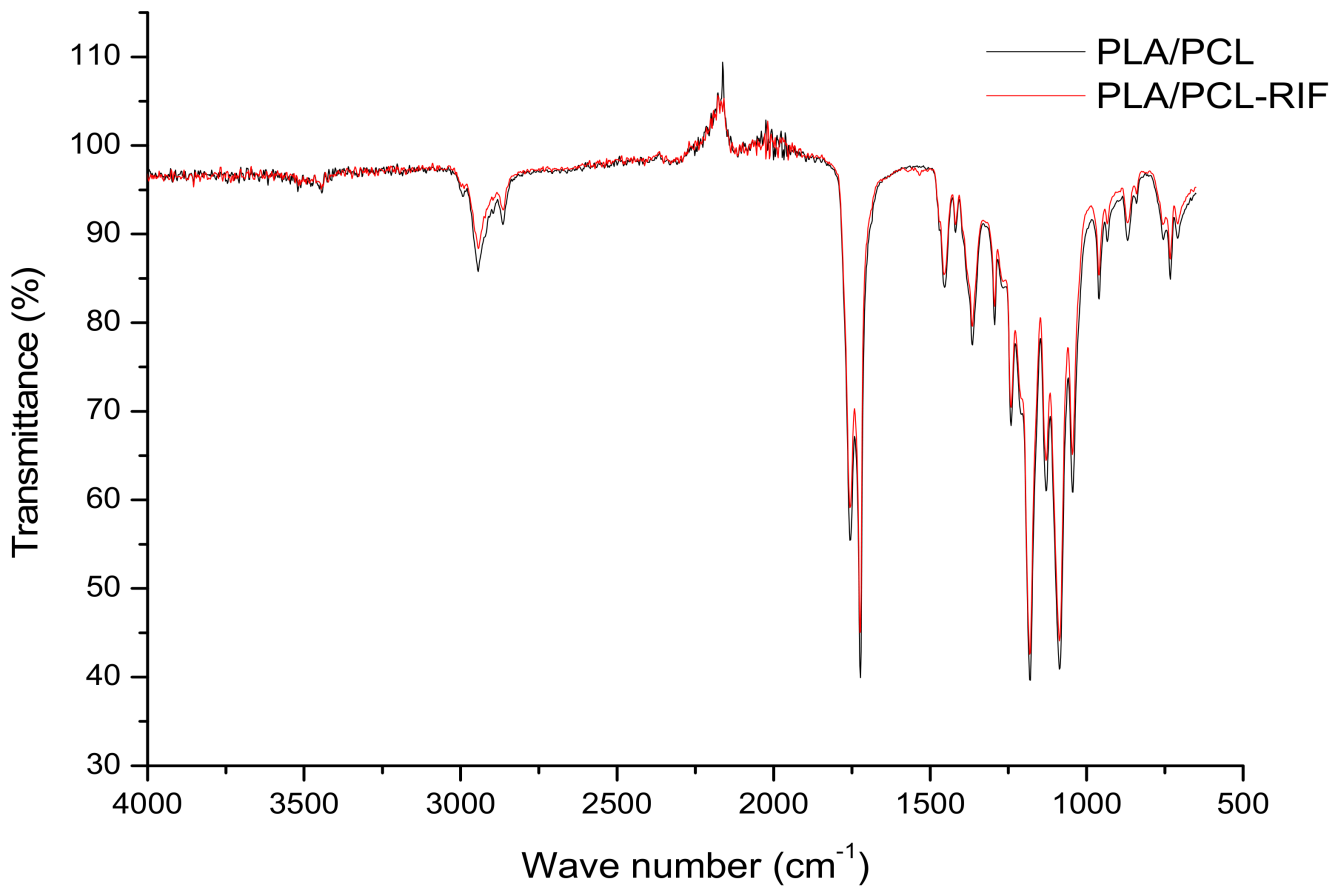
FTIR spectra of drug-free PLA/PCL and rifampicin-loaded PLA/PCL–RIF electrospun nanofibers.

The FTIR spectra of the electrospun dressings are shown in Figure 2. The absorption band observed at 2943 cm^−1^ was attributed to the asymmetric stretching vibration of the methyl (–CH_3_) group, which is present in both PLA and rifampicin. The intensity of this band is associated with the relative abundance of these functional groups in the nanofiber formulation. A less intense band at 2865 cm^−1^ was assigned to methylene (–CH_2_–) stretching vibrations, mainly associated with PCL and PLA. At lower wavenumbers, characteristic carbonyl (C=O) stretching bands were observed at 1755 cm^−1^ for PLA and 1725 cm^−1^ for PCL. In addition, the fingerprint region showed the characteristic absorption bands of these polymers, with prominent peaks at 1080 cm^−1^ for PLA and 1240 cm^−1^ for PCL. Rifampicin incorporation did result in substantial changes in the FTIR profile of the nanofibers, as the spectra of PLA/PCL and PLA/PCL–RIF remained highly similar [45,46,47,48]. This behavior is likely related to the relatively low rifampicin concentration incorporated into the fibers, which may have limited its spectral contribution relative to that of the polymeric matrix [49,50]. Collectively, the FTIR results suggest that rifampicin incorporation did not induce detectable chemical modifications in the polymeric matrix under the electrospinning conditions employed.

### 3.3. Evaluation of Molecular Ordering by X-Ray Diffraction (XRD)

**Figure 3:**
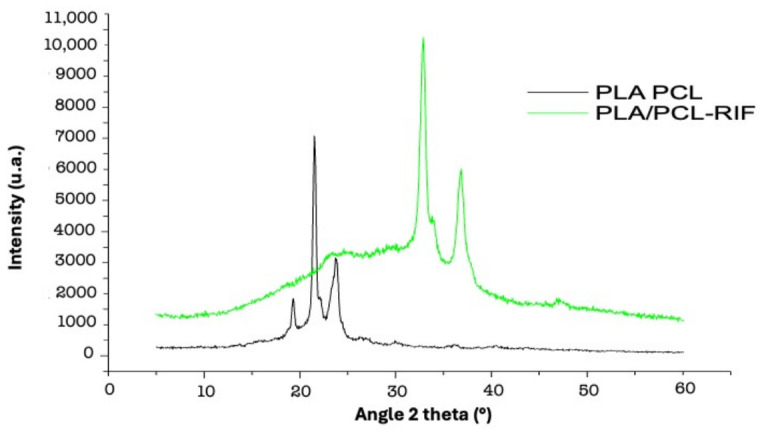
X-ray diffraction (XRD) patterns of electrospun PLA/PCL and rifampicin-loaded PLA/PCL–RIF nanofibers.

X-ray diffraction analysis was performed to assess the molecular ordering and crystalline structure of the electrospun nanofibers. As shown in Figure 3, the drug-free PLA/PCL nanofibers exhibited a partially ordered structure, with diffraction peaks centered at 19.21°, 21.52°, and 23.77° (2θ), indicating the presence of crystalline domains within the polymeric matrix. These reflections are consistent with the semicrystalline nature of both PLA and PCL, whose diffraction patterns typically exhibit characteristic crystalline peaks superimposed on a broader amorphous halo.

The diffraction pattern of the PLA/PCL blend suggests the coexistence of crystalline contributions from both polymers. The reflections observed at approximately 21.5° and 23.8° are consistent with the characteristic crystalline planes commonly reported for PCL, whereas the reflection centered near 19.2° has been associated with ordered regions of PLA, although overlap between both polymer contributions cannot be excluded. Since PLA and PCL are generally considered poorly miscible in the solid state, the resulting diffractogram can be interpreted as the superposition of the individual diffraction features of each polymer, rather than the formation of a new crystalline phase. This observation supports the coexistence of phases after solvent evaporation during electrospinning.

Compared with the drug-free membranes, the PLA/PCL–RIF nanofibers exhibited attenuation of the diffraction peak centered near 19° together with slight shifts in the remaining crystalline reflections. Rather than indicating the formation of a new crystalline phase, these changes suggest that rifampicin incorporation modified the molecular packing of the polymer chains during electrospinning. Rifampicin is a relatively bulky molecule containing multiple hydroxyl and carbonyl functional groups that can establish intermolecular interactions with the polyester matrix. These interactions are expected to hinder chain rearrangement during solvent evaporation and subsequent crystallization, thereby reducing the development of ordered crystalline domains and increasing the amorphous fraction of the electrospun membrane. Similar effects have been reported for drug-loaded electrospun polyester systems, where the incorporated drug acts as a physical obstacle to crystal growth without altering the chemical structure of the polymer matrix [51,52].

The slight displacement of the diffraction peaks may also reflect local variations in the interplanar spacing caused by changes in chain packing after drug incorporation. Such peak shifts have been associated with modifications in molecular organization arising from polymer–drug interactions rather than the formation of additional crystalline phases [53].

Despite these structural changes, the persistence of the main crystalline reflections indicates that the PLA/PCL–RIF membranes retained their overall semicrystalline character. This partial preservation of crystallinity is advantageous because it contributes to maintaining the structural integrity of the electrospun membranes while introducing a larger amorphous fraction that may facilitate drug diffusion and sustained release. Furthermore, the increased amorphous character suggested by the XRD results is consistent with the slight reduction in mechanical performance observed after rifampicin incorporation, since lower chain packing efficiency generally decreases stress transfer within electrospun fibrous networks [46,52].

### 3.4. Mechanical Performance of Electrospun Dressings After Accelerated Temperature–Humidity Aging

The mechanical performance of the electrospun dressings after accelerated temperature–humidity aging is summarized in Table 1. Tensile testing was performed to evaluate Young’s modulus (E), ultimate tensile strength (UTS), stress at break (σb), and elongation at break (εb), which are key parameters for assessing the structural integrity and handling capability of the dressings after environmental exposure. Among the evaluated formulations, PLA/PCL/RIF (I) exhibited the highest mechanical strength, with values of 2.54 MPa for Young’s modulus, 2.14 MPa for both UTS and stress at break, and an elongation at break of 1.03%. In contrast, PLA/PCL/RIF (II) showed lower tensile performance, with a UTS of 1.50 MPa, a stress at break of 1.49 MPa, and a lower elongation at break (0.65%). Although both formulations displayed similarly low deformability, the reduction in tensile strength observed for PLA/PCL/RIF (II) suggests susceptibility to brittle failure after climatic aging. The elongation values observed in both formulations indicate a predominantly brittle mechanical response, which is consistent with the semicrystalline character of the PLA/PCL matrix and the structural changes induced by the temperature–humidity cycling treatment. The results suggest that rifampicin incorporation, together with moisture uptake during aging, is associated with the formation of microstructural heterogeneities within the fibrous network, which can influence stress distribution and contribute to earlier fracture under tensile loading. Thus, the results indicate that PLA/PCL–RIF dressings retain sufficient tensile strength after accelerated temperature–humidity aging, supporting their mechanical stability under adverse environmental conditions. This preserved strength enables reliable handling, placement, and removal without structural failure, which is essential for routine clinical or at-home use. In addition, their inherent rigidity contributes to dimensional stability and promotes sustained contact with the skin surface, both of which are critical for achieving consistent and controlled transdermal drug delivery. These characteristics are particularly advantageous for maintaining uniform drug release over time and minimizing the risk of deformation or detachment during application.

### 3.5. Cytotoxicity and Biocompatibility of PLA/PCL–RIF Dressings in Human Cells

**Figure 4:**
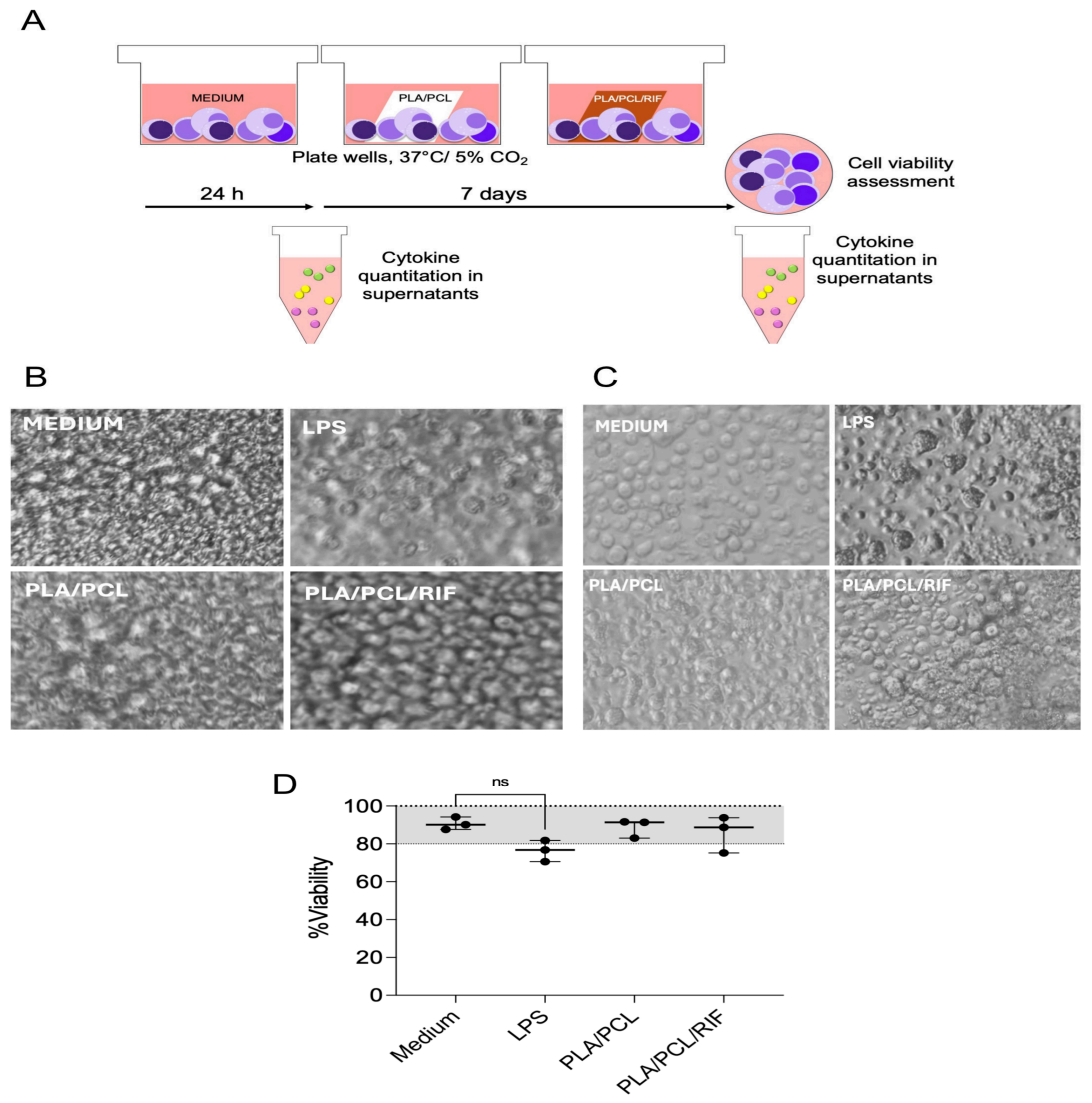
Cytotoxicity assessment of PLA/PCL and PLA/PCL–RIF dressings in primary human leukocytes. (**A**) In vitro exposure model of peripheral blood leukocytes to the electrospun dressings. Peripheral blood mononuclear cells (PBMCs) from healthy donors were incubated at 37 °C in a humidified atmosphere containing 5% CO_2_ in the presence of medium alone (negative control), LPS (positive control), PLA/PCL, or PLA/PCL–RIF dressings. Culture supernatants were collected after (**B**) 24 h and (**C**) 7 days of incubation. Representative optical micrographs of leukocyte cultures after 24 h and 7 days of exposure. Images correspond to one representative donor out of seven analyzed. (**D**) Cell viability was determined by trypan blue exclusion after 7 days of culture. Data from three donors are shown as individual values together with the median and interquartile range. The shaded area indicates the safety threshold. n = 3; ns, not significant; repeated-measures one-way ANOVA with Geisser–Greenhouse correction followed by Dunnett’s multiple comparison test.

The dressing was prepared to yield 1 cm^2^ pieces containing rifampicin within the range of concentrations safe for in vitro experimental procedures involving mammalian cells or tissue [54,55]; still, we determined the cytotoxicity and biocompatibility of the dressings to account for the chemicals involved in their preparation. The cytotoxicity and biocompatibility of the electrospun dressings were evaluated by exposing primary human leukocytes to 1 cm^2^ sections of PLA/PCL and PLA/PCL–RIF for 7 days (Figure 4A). Culture supernatants were collected after 24 h to assess the early cellular response to material exposure, and fresh medium was subsequently added. At the end of the 7-day incubation period, cell morphology, viability, and cytokine production were evaluated. Unstimulated cells cultured in medium alone served as the negative control (MEDIUM), whereas lipopolysaccharide (LPS) was used as the positive control for inflammatory activation.

After 24 h of culture, control cells exhibited an intact monolayer covering the bottom of the well (Figure 4B). Neither PLA/PCL nor PLA/PCL–RIF altered cell morphology or cellular distribution at this time point. After 7 days of incubation, adherent cells with macrophage-like morphology predominated in all experimental conditions (Figure 4C), and no evident morphological alterations were observed in response to either dressing formulation. These observations were consistent with the cell viability results (Figure 4D), which remained above the 80% threshold commonly considered indicative of non-cytotoxic behavior (88.7 ± 2.8 and 85.8 ± 5.5, respectively). Accordingly, both PLA/PCL and PLA/PCL–RIF dressings were classified as non-cytotoxic under the evaluated conditions. As expected, LPS exposure reduced cell viability, although this effect did not reach statistical significance.

**Figure 5:**
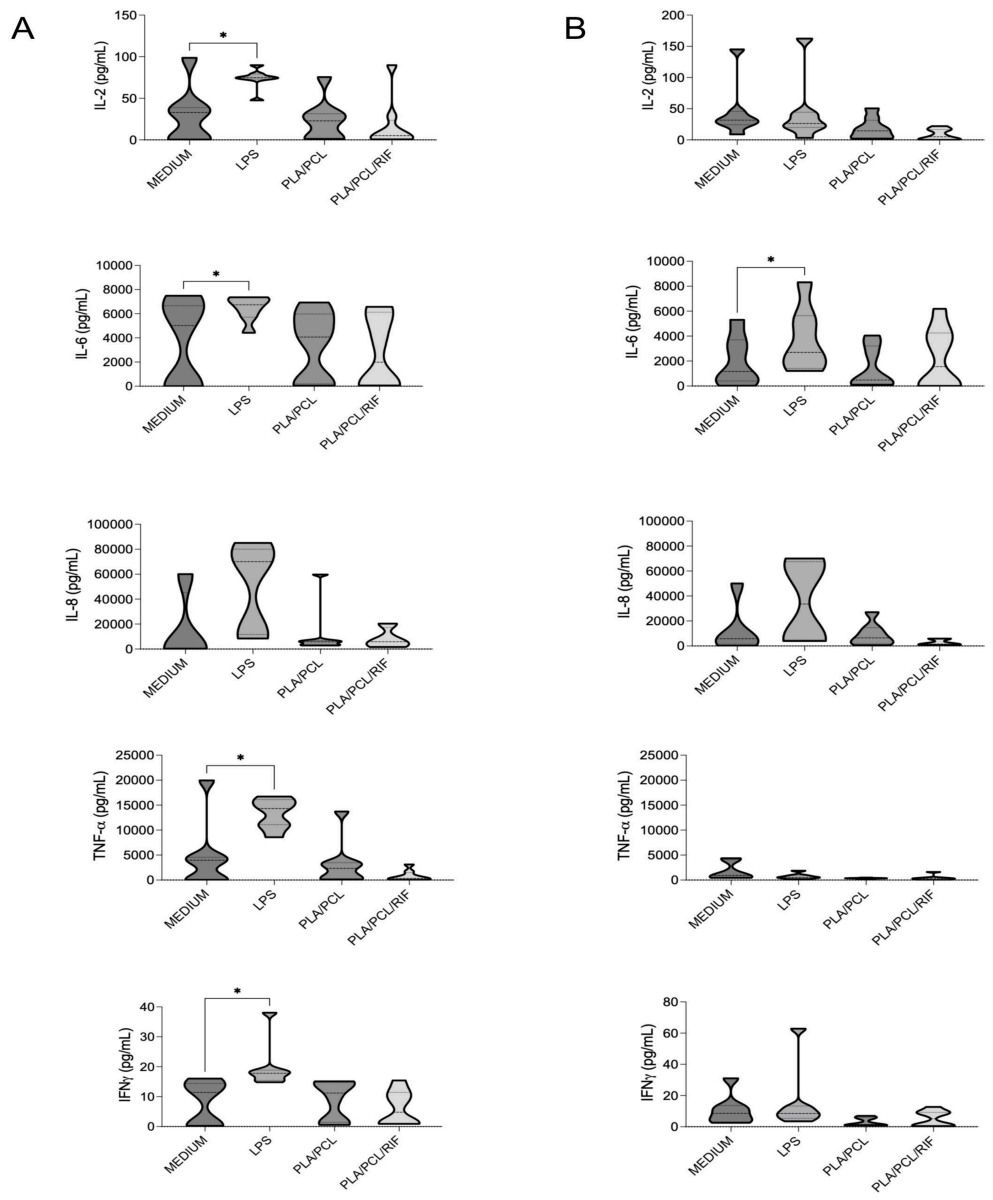
Pro-inflammatory cytokine production by primary human leukocytes exposed to PLA/PCL and PLA/PCL–RIF dressings. Peripheral blood mononuclear cells were incubated at 37 °C in a humidified atmosphere containing 5% CO_2_ in the presence of medium (negative control), LPS (positive control), PLA/PCL, or PLA/PCL–RIF. Cytokine production was quantified at early (24 h, (**A**)) and late (7 days, (**B**)) time points using Luminex technology. Data are presented as violin plots showing the distribution of values; n = 7, * *p* < 0.05, Wilcoxon rank-sum test.

To further assess biocompatibility, cytokine production was quantified in culture supernatants (Figure 5A,B). At 24 h, cells cultured in medium alone produced only basal physiological levels of cytokines, whereas LPS-stimulated cells showed a marked inflammatory response, with significantly increased production of the pro-inflammatory cytokines IL-2, IL-6, TNF-α, and IFN-γ (Figure 5A), as well as lower but significant increases in IL-10, IL-4, and GM-CSF (Figure 5A). In contrast, neither PLA/PCL nor PLA/PCL–RIF induced significant production of pro-inflammatory or anti-inflammatory cytokines. After 7 days of culture, cytokine quantification was repeated to assess possible delayed incompatibility effects. Under these conditions, only IL-6 remained significantly elevated in the LPS-treated group (Figure 5B), whereas both PLA/PCL and PLA/PCL–RIF continued to show cytokine levels comparable to the negative control (Figure 5B). Overall, these findings indicate that the rifampicin-loaded dressing is both safe and biocompatible in primary human leukocyte cultures.

### 3.6. Transdermal Permeation of Rifampicin: Proof-of-Concept Evaluation

**Figure 6:**
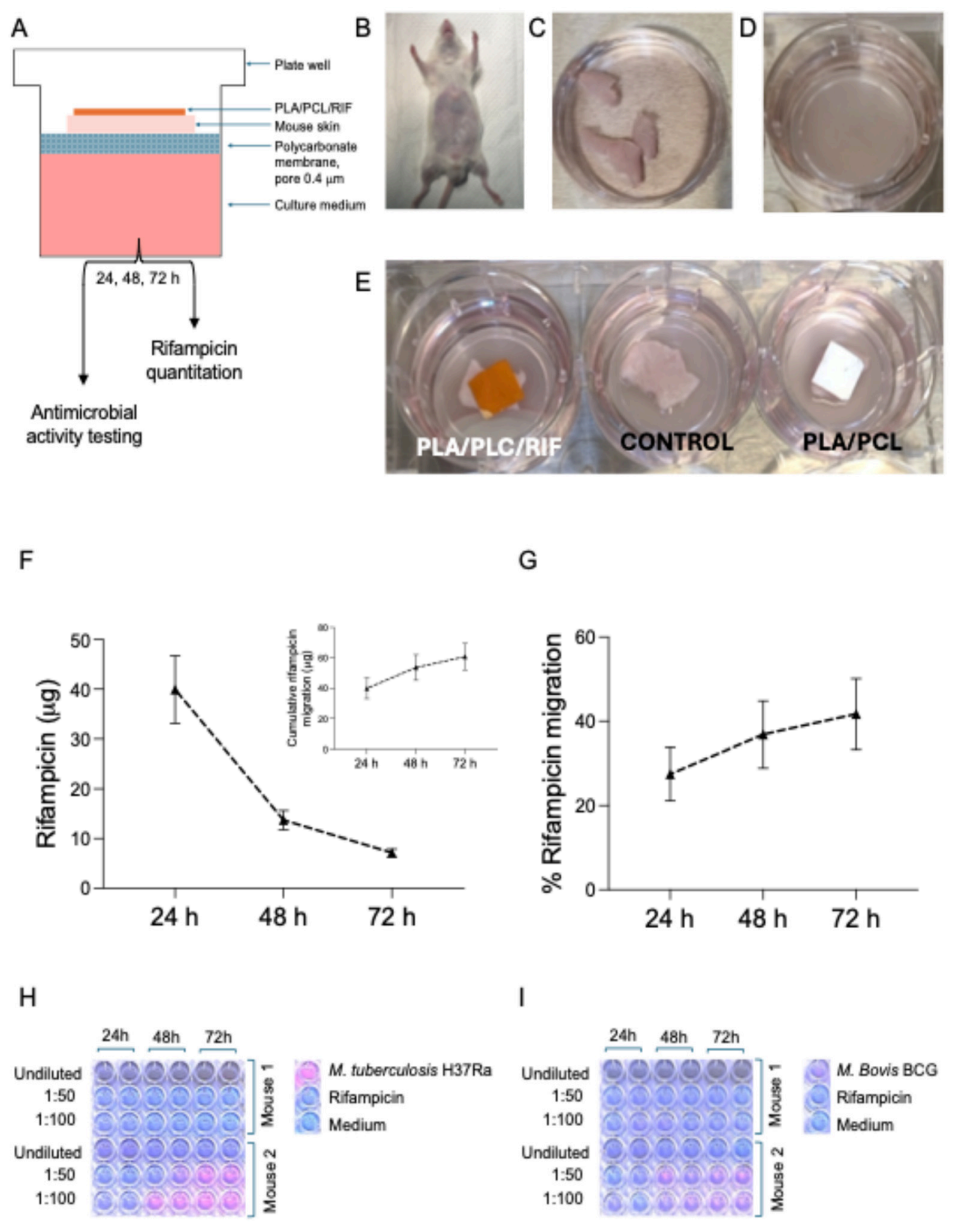
Proof-of-concept evaluation of rifampicin transdermal delivery from PLA/PCL–RIF dressings. (**A**) An in vitro model was used to evaluate rifampicin diffusion through mouse skin. (**B**–**E**) Drug-free PLA/PCL and rifampicin-loaded PLA/PCL–RIF dressings (1 cm^2^) were placed on approximately 1 cm^2^ sections of abdominal skin obtained from BALB/c mice (n = 4). Rifampicin permeating through the skin was quantified in the culture medium at 24, 48, and 72 h by HPLC-DAD (**F**); the inset shows cumulative rifampicin. The percentage of rifampicin permeated was calculated at each 24 h interval; cumulative permeation percentages are shown, n = 4, and means ± SEM are depicted (**G**). REMA was used to evaluate the antimicrobial activity of recovered culture media against *Mycobacterium tuberculosis* H37Ra (**H**) and *Mycobacterium bovis* BCG (**I**). Pure rifampicin at 200 ng/mL was used as a positive control, and 7H9 medium alone served as a negative control for bacterial killing. Untreated bacterial cultures were used as a positive control for resazurin reduction. Representative images from two mice are depicted.

A transdermal diffusion was evaluated using the electrospun dressings in an in vitro mouse skin model (Figure 6A–E). Abdominal skin sections (~1 cm^2^) obtained from four euthanized mice were mounted on polycarbonate transwell inserts, and 1 cm^2^ sections of PLA/PCL or PLA/PCL–RIF dressings were placed in direct contact with the skin surface. The dressings contained 153 ± 17 mg of RIF. Culture medium was collected and replaced every 24 h, allowing rifampicin (RIF) permeation to be quantified in consecutive 24 h intervals rather than as cumulative release.

The amounts of RIF that migrated through the skin decreased from 40 ± 7 µg/cm^2^ at 24 h to 14 ± 2 at 48 h and 7 ± 1 at 72 h, indicating a time-dependent release with decreasing flux over time (Figure 6F). Because the measurements were performed every 24 h independently, the inset depicts the cumulative rifampicin released through the skin, which amounted to 61 ± 9 mg/cm^2^ of skin at 72 h. No RIF was detected in the drug-free PLA/PCL control samples at any time point, confirming the absence of chromatographic interference from the polymeric matrix. To assess the rate of drug migration, we calculated the percentage of rifampicin relative to the concentration remaining in the dressing (Figure 6G). At 24 h, 47 ± 6% of the rifampicin was released, whereas 37 ± 8% was released at 48 h, and 42 ± 8% was released at 72 h. No significant differences were detected, suggesting steady cumulative permeation and sustained transdermal delivery of RIF over at least 72 h, without evidence of abrupt burst release or uncontrolled depletion (Figure 6G).

Other transdermal rifampicin delivery systems have been reported, but their drug-permeation efficiency has been limited. In a nanoemulsion formulation, approximately 10% of rifampicin diffused across rat abdominal skin within 24 h, increasing to about 15% after incorporating a transdermal permeation enhancer [29]. Likewise, a cellulose-based dressing showed no detectable rifampicin permeation after 24 h of exposure in a neonatal porcine skin model; however, the addition of hydrogel microneedles increased skin permeation to approximately 19% [31]. In comparison, our device achieved a mean transdermal delivery of 27.5% during the first 24 h, reaching a cumulative 41.7% by day 3. Although direct comparisons should be interpreted with caution because of differences in formulation design, experimental conditions, and skin models, these results suggest that our platform provides more efficient and sustained transdermal delivery of rifampicin than previously reported systems.

To assess whether RIF retained its antimicrobial activity after electrospinning, skin permeation, and recovery from the culture medium, the recovered samples were further evaluated using the REMA against rifampicin-sensitive mycobacteria (Figure 6H,I). In this assay, viable and metabolically active bacteria reduce blue resazurin to pink resorufin, whereas maintenance of the blue signal indicates growth inhibition. Pure RIF at 200 ng/mL was used as a positive control, and 7H9 medium alone served as the negative control. Since the concentrations of RIF recovered from the transdermal system exceeded the minimum levels required for biological activity, 1:50 and 1:100 dilutions were also tested. In all cases, undiluted samples recovered from the system effectively inhibited the growth of *Mycobacterium tuberculosis* H37Ra (Figure 6H) and *Mycobacterium bovis* BCG (Figure 6I). The activity decreased at later time points after dilution, particularly for the 48 and 72 h samples, where the effective drug concentration became limited.

In summary, the present study demonstrates that electrospun PLA/PCL nanofiber dressings can effectively incorporate rifampicin and enable its sustained transdermal delivery while preserving its biological activity. The integration of physicochemical characterization, mechanical evaluation, and biological validation provides a comprehensive assessment of the system as a potential drug delivery platform. The PLA/PCL–RIF dressing enabled sustained transdermal delivery of biologically active rifampicin for at least 72 h in this proof-of-concept model. From a therapeutic perspective, sustained transdermal delivery of rifampicin may reduce dosing frequency and improve treatment adherence. Although tuberculosis treatment typically requires multidrug regimens, rifampicin is also used in preventive therapy for selected individuals at high risk of progression from latent to active infection [56,57]. In this context, the present findings support the potential of these electrospun dressings for sustained antibiotic delivery in preventive therapy. Further studies are required to validate their performance under clinically relevant conditions.

## 4. Conclusions

This study presents a reproducible method for fabricating electrospun polymeric nanofiber dressings that can incorporate rifampicin (RIF). The PLA/PCL-based systems enabled efficient drug encapsulation and yielded continuous nanofibrous structures with homogeneous morphology and low defect density, with characteristics suitable for transdermal drug delivery applications. FTIR analysis confirmed the successful incorporation of rifampicin into the polymeric matrix without evidence of new chemical bond formation or structural alterations attributable to the electrospinning process.

Rifampicin was incorporated into the electrospun PLA/PCL dressings with loading efficiencies above 98%. The resulting systems did not induce significant cytotoxic or pro-inflammatory effects in primary human immune cells but demonstrated sustained transdermal permeation of biologically active rifampicin for at least 72 h in a proof-of-concept mouse skin model. Importantly, the drug recovered after skin permeation retained antimycobacterial activity against *Mycobacterium tuberculosis* H37Ra and *Mycobacterium bovis* BCG, indicating that neither the fabrication process nor the transdermal delivery conditions compromise its biological activity. Rifampicin-loaded PLA/PCL dressings retained sufficient tensile strength after accelerated temperature–humidity aging, preserving their structural integrity and suggesting reliable handling by the recipients.

Taken together, these findings position the electrospun PLA/PCL–RIF dressings as promising platforms for the controlled transdermal delivery of antibiotics. At the same time, the results highlight the importance of further optimizing formulation design, structural stability, and long-term performance under physiologically relevant conditions to advance these systems toward therapeutic application.

## Figures and Tables

**Table 1:** Mechanical properties of electrospun PLA/PCL–RIF nanofibers after temperature–humidity aging.

Sample	E (MPa)	UTS (MPa)	σb (MPa)	εb %
PLA/PCL/RIF (I)	2.54 ± 1.03	2.14 ± 0.68	2.14 ± 0.69	1.03 ± 0.31
PLA/PCL/RIF (II)	2.52 ± 0.48	1.50 ± 0.44	1.49 ± 0.43	0.65 ± 0.10

Values are expressed as mean ± standard deviation (n = 3). E: Young’s modulus; UTS: ultimate tensile strength; σb: stress at break; εb: elongation at break. PLA: polylactic acid; PCL: poly(ε-caprolactone); RIF: rifampicin.

## Data Availability

The original contributions presented in this study are included in the article. Further inquiries can be directed to the corresponding authors.

## Abbreviations

The following abbreviations are used in this manuscript:

ADC	Albumin Dextrose Catalase Supplement
ATR	Attenuated Total Reflectance
BCG	Bacillus Calmette–Guérin
CO_2_	Carbon Dioxide
DAD	Diode Array Detector
DMF	N,N-Dimethylformamide
DSC	Differential Scanning Calorimetry
E	Young’s Modulus
FTIR	Fourier Transform Infrared Spectroscopy
GM-CSF	Granulocyte-Macrophage Colony-Stimulating Factor
HPLC-DAD	High-Performance Liquid Chromatography with Diode Array Detection
HR	Relative Humidity
IEC	International Electrotechnical Commission
IFN-γ	Interferon Gamma
IL	Interleukin
LPS	Lipopolysaccharide
MDR	Multidrug Resistance
PBS	Phosphate-Buffered Saline
PBMCs	Peripheral Blood Mononuclear Cells
PCL	Poly(ε-caprolactone)
PLA	Poly(lactic acid)
REMA	Resazurin Microtiter Assay
RIF	Rifampicin
RPMI-1640	Roswell Park Memorial Institute 1640 Medium
SEM	Scanning Electron Microscopy
TB	Tuberculosis
TNF-α	Tumor Necrosis Factor Alpha
UTS	Ultimate Tensile Strength
XRD	X-ray Diffraction
εb	Elongation at Break
σb	Stress at Break
